# Species Designations Belie Phenotypic and Genotypic Heterogeneity in Oral Streptococci

**DOI:** 10.1128/mSystems.00158-18

**Published:** 2018-12-18

**Authors:** Irina M. Velsko, Brinta Chakraborty, Marcelle M. Nascimento, Robert A. Burne, Vincent P. Richards

**Affiliations:** a Department of Biological Sciences, Clemson University, Clemson, South Carolina, USA; b Department of Oral Biology, College of Dentistry, University of Florida, Gainesville, Florida, USA; c Department of Restorative Dental Sciences, Division of Operative Dentistry, College of Dentistry, University of Florida, Gainesville, Florida, USA; University of California, San Francisco

**Keywords:** *Streptococcus*, genomics, oral microbiology, phylogenetic analysis, variable phenotypes

## Abstract

Health-associated oral *Streptococcus* species are promising probiotic candidates to protect against dental caries. Ammonia production through the arginine deiminase system (ADS), which can increase the pH of oral biofilms, and direct antagonism of caries-associated bacterial species are desirable properties for oral probiotic strains. ADS and antagonistic activities can vary dramatically among individuals, but the genetic basis for these differences is unknown. We sequenced whole genomes of a diverse set of clinical oral *Streptococcus* isolates and examined the genetic basis of variability in ADS and antagonistic activities. A total of 113 isolates were included and represented 10 species: Streptococcus australis, A12-like, S. cristatus, S. gordonii, S. intermedius, S. mitis, S. oralis including S. oralis subsp. *dentisani*, S. parasanguinis, S. salivarius, and S. sanguinis. Mean ADS activity and antagonism on Streptococcus mutans UA159 were measured for each isolate, and each isolate was whole genome shotgun sequenced on an Illumina MiSeq. Phylogenies were built of genes known to be involved in ADS activity and antagonism. Several approaches to correlate the pan-genome with phenotypes were performed. Phylogenies of genes previously identified in ADS activity and antagonism grouped isolates by species, but not by phenotype. A genome-wide association study (GWAS) identified additional genes potentially involved in ADS activity or antagonism across all the isolates we sequenced as well as within several species. Phenotypic heterogeneity in oral streptococci is not necessarily reflected by genotype and is not species specific. Probiotic strains must be carefully selected based on characterization of each strain and not based on inclusion within a certain species.

**IMPORTANCE** Representative type strains are commonly used to characterize bacterial species, yet species are phenotypically and genotypically heterogeneous. Conclusions about strain physiology and activity based on a single strain therefore may be inappropriate and misleading. When selecting strains for probiotic use, the assumption that all strains within a species share the same desired probiotic characteristics may result in selection of a strain that lacks the desired traits, and therefore makes a minimally effective or ineffective probiotic. Health-associated oral streptococci are promising candidates for anticaries probiotics, but strains need to be carefully selected based on observed phenotypes. We characterized the genotypes and anticaries phenotypes of strains from 10 species of oral streptococci and demonstrate poor correlation between genotype and phenotype across all species.

## Abstract (precis)

Representative type strains are commonly used to characterize bacterial species, yet species are phenotypically and genotypically heterogeneous. Conclusions about strain physiology and activity based on a single strain therefore may be inappropriate and misleading. When selecting strains for probiotic use, the assumption that all strains within a species share the same desired probiotic characteristics may result in selection of a strain that lacks the desired traits, and therefore makes a minimally effective or ineffective probiotic. Health-associated oral streptococci are promising candidates for anticaries probiotics, but strains need to be carefully selected based on observed phenotypes. We characterized the genotypes and anticaries phenotypes of strains from 10 species of oral streptococci and demonstrate poor correlation between genotype and phenotype across all species.

## INTRODUCTION

Dental caries is a significant health problem and the most common oral infectious disease, causing substantial morbidity worldwide. Caries develop when the tooth enamel is demineralized through successive exposure to low pH, a condition driven by fermentation of dietary carbohydrates into organic acids by acidogenic oral bacterial species. Treatment for caries can be expensive, and disease prevention is a major goal of oral health care research.

The use of orally administered probiotic species is gaining popularity as a strategy for maintaining oral health. This involves introducing bacterial strains to the oral cavity with the goal of promoting growth and metabolic activity of a health-associated oral biofilm while suppressing growth and metabolic activity of disease-associated species. Several studies have demonstrated successful *in vivo* and *in vitro* application of dairy product-derived oral probiotic species, predominantly lactobacilli, highlighting the potential of probiotics in oral health care. Select strains of lactobacilli inhibit growth and biofilm formation of caries-associated species Streptococcus mutans and Candida albicans in culture (1–3), which is a prime caries prevention strategy. *In vitro* biofilm growth assays demonstrated that strains of *Lactobacillus*, *Lactococcus*, and *Streptococcus* can integrate into saliva-derived or defined-species biofilms and are maintained in the biofilms over several days (4–8). However, an *in vivo* study reported that no probiotic lactobacilli were detected in dental plaque of individuals after 8-day treatment with fermented milk (9), so the method by which such probiotic strains act on the biofilm *in vivo* needs to be investigated further.

In addition to food-derived probiotic strains, there are many bacterial species in dental plaque that are associated with health, which may be mined for probiotic potential. These oral species have the advantage of being adapted to growth in the mouth and the oral biofilm, and they may offer more sustainable and longer-term probiotic benefits than species from external sources like dairy products. In particular, several *Streptococcus* species, including S. gordonii, S. sanguinis, and S. salivarius are associated with oral health (10–12), and *S. salivarius* K12 has been adapted as a probiotic for pharyngitis/tonsillitis (13), halitosis (14), and otitis media (15).

Buffering biofilm pH through ammonia production is a promising health-associated activity of oral streptococci (10). The arginine deiminase system (ADS) is a dominant method used by streptococci to produce ammonia from arginine. This pathway has been extensively characterized in S. gordonii (16–19) and consists of an operon containing five genes encoding structural proteins, *arcA* (arginine deiminase), *arcB* (ornithine carbamoyltransferase), *arcC* (carbamate kinase), *arcD* (arginine-ornithine antiporter), and *arcT* (putative transaminase or peptidase), and two regulatory genes immediately downstream that are cotranscribed in the opposite direction from the operon, *arcR* and *queA,* which are essential for optimal ADS activity in S. gordonii (16). Additionally, upstream of *arcA* is *flp*, another regulatory element involved in ADS activity (17). Expression of the ADS operon is regulated by environmental factors, including the presence of arginine (18), sugar carbon source (17), and the presence of oxygen (18, 20). Strains with defective ADS expression or regulation are more sensitive to pH-induced killing (19), suggesting that this pathway is important for maintaining health-associated species in the presence of acidogenic species.

The use of arginine-containing toothpaste and mint prebiotics to boost ADS activity in plaque and protect against caries development and progression is also a promising method to promote health-associated activity in oral biofilms (21–24). As arginine directly affects growth and pathogenesis of S. mutans (25), developing probiotics that target arginine metabolism may be especially effective in preventing caries (26). Arginine catabolism is clinically relevant to caries development. Clinical studies have shown that ADS activity is higher in plaque and saliva of patients who have never had caries than patients with active caries, both in adults (27) and children (28). Further, Nascimento et al. (27) found an inverse relationship between ADS activity and abundance of S. mutans in plaque samples. Unexpectedly, they found no correlations between the abundance of health-associated *Streptococcus* species and ADS activity level, while some plaque samples from caries sites had high ADS activity (28). They concluded that there must be more to ADS activity than simply the presence or abundance of health-associated streptococci.

Recent phenotypic characterization of ADS activity in a variety of oral *Streptococcus* species grown in different conditions (arginine availability, pH, carbohydrate source, and oxygen tension) showed substantial variation in activity within and between species and within growth conditions (29). This confirmed the conclusion of Nascimento et al. (28) that no health-associated *Streptococcus* species are collectively associated with ADS activity; rather, ADS activity is highly strain specific. In addition, these clinical isolates had a range of ability to antagonize growth of S. mutans (29), also a desirable trait in a probiotic strain. The genetic basis for variability in ADS activity and S. mutans antagonism has not yet been examined, but it may provide insight for probiotic development. Rational selection of probiotic strains is particularly important because of the genotypic and phenotypic heterogeneity within oral *Streptococcus* species. Here we examined the probiotic properties and genome composition of a wide variety of oral *Streptococcus* species isolated from dental plaque. We show substantial phenotypic and genotypic heterogeneity of all species examined, which has implications for targeted probiotic strain selection.

## RESULTS

### Species assignments.

A total of 113 *Streptococcus* species were isolated from supragingival dental plaque samples, characterized for ADS activity and antagonism on and by Streptococcus mutans UA159, and whole genome shotgun sequenced. Nine species were identified by 16S rRNA gene sequencing of 106 isolates, and seven isolates could not be identified to the species level. Core genome analysis confirmed that we characterized and sequenced 2 Streptococcus australis isolates, 2 A12-like isolates, 11 S. cristatus isolates, 17 S. gordonii isolates, 11 S. intermedius isolates, 27 S. mitis isolates, 8 S. oralis isolates, 6 S. oralis subsp. *dentisani* isolates, 25 S. sanguinis isolates, 1 isolate each of S. parasanguinis and S. salivarius, and 2 isolates for which a species could not be identified as they grouped with the S. mitis*/*S. oralis complex in the phylogeny (see Fig. S1 and Table S1 in the supplemental material). Given that previous work has placed S. oralis subsp. *dentisani* as a distinct subclade of S. oralis (30), we performed all analyses by both grouping all isolates together and keeping them as separate groups. Likewise, the *S. australis* isolates and A12-like isolates were grouped together for all analyses because we had only two isolates of each and they were more closely related to each other than to other *Streptococcus* species (31). Based on the core genome phylogeny of our isolates, the phylogenetic relationships of the species we sequenced follow the branching patterns reported for these species within the genus *Streptococcus* (32) with the exception that S. oralis, S. mitis, and S. oralis subsp. *dentisani* are intermixed within their clade with no clear species groupings (Fig. S1).

10.1128/mSystems.00158-18.1 TABLE S1 List of isolates sequenced in this study and their biochemical characteristics. Download Table S1, XLSX file, 0.03 MB. Copyright © 2018 Velsko et al. 2018 Velsko et al. This content is distributed under the terms of the Creative Commons Attribution 4.0 International license.

10.1128/mSystems.00158-18.4 FIG S1 Phylogenetic relationship of isolates sequenced for this study. A maximum likelihood phylogeny based on a core set of 425 putatively nonrecombinant genes is shown. Download FIG S1, PDF file, 0.2 MB. Copyright © 2018 Velsko et al. 2018 Velsko et al. This content is distributed under the terms of the Creative Commons Attribution 4.0 International license.

### Heterogeneity of arginine deiminase activity and antagonism toward S. mutans within diverse *Streptococcus* species.

**FIG 1:**
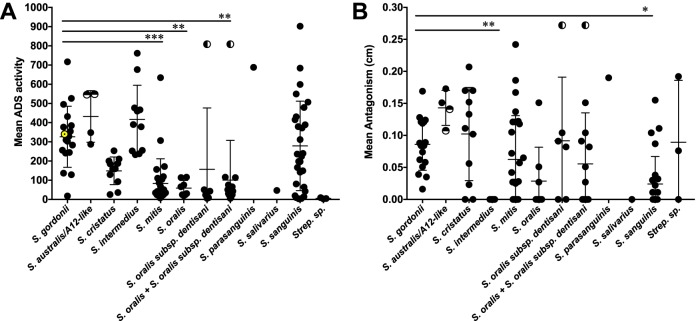
Phenotypic diversity within diverse clinical oral *Streptococcus* isolates. (A) Mean ADS activity of each isolate included in this study. The mean ADS activity of S. gordonii DL1 is included for reference as a yellow circle with a black dot in its center. (B) Mean antagonism of S. mutans UA159 of each isolate included in this study. The half-filled circles in the *S. australis*/A12-like column indicate the A12-like isolates. The half-filled circles in the S. oralis subsp. *dentisani* and S. oralis + S. oralis subsp. *dentisani* columns indicate the same isolate. Values that are significantly different are indicated by bars and asterisks as follows: *, *P* < 0.05; **, *P* < 0.01; ***, *P* < 0.001.

Phenotypic heterogeneity was shown by the range of ADS activity and antagonism toward Streptococcus mutans within strains of each species (Fig. 1 and Table 1). As arginine deiminase activity was first described in S. gordonii DL1, we used S. gordonii as the reference group for our statistical tests. Streptococcus mitis, S. oralis, and S. oralis plus S. oralis subsp. *dentisani* each had significantly lower ADS activity by one-way ANOVA than S. gordonii did (Fig. 1A and Table 1). The large standard deviations demonstrate substantial species phenotypic diversity, particularly in S. gordonii and S. sanguinis. A single isolate of S. oralis subsp. *dentisani* had exceptionally high ADS activity for the species (half-filled circle in Fig. 1) and was responsible for that group’s large standard deviation. The *S. australis* isolates and A12-like isolates separated into two clusters, with the A12-like isolates (half-filled circles in Fig. 1A) exhibiting higher average ADS activity than the *S. australis* isolates (filled circles).

**TABLE 1:** Mean arginine deiminase activity and S. mutans antagonism of diverse *Streptococcus* species

Species	No. of isolates	ADS activity (nmol/min/mg of protein)^*a*^	Antagonism (mm)^*a*^
S. australis/A12-like	4	432 ± 116	1.4 ± 0.2
S. cristatus	11	149 ± 68	1.0 ± 0.7
S. gordonii	17	325 ± 159	0. 9 ± 0.4
S. intermedius	11	417 ± 169	0 ± 0
S. mitis	27	83.7 ± 125***	0.6 ± 0.7
S. oralis	8	58.8 ± 37**	0.28 ± 0.5
S. oralis subsp. *dentisani*	6	156 ± 291	0.91 ± 0.9
S. oralis + S. oralis subsp. *dentisani*	14	100 ± 199**	0.56 ± 0.8
S. parasanguinis	1	688	1.9
S. salivarius	1	46.8	0
S. sanguinis	25	311 ± 220	0.22 ± 0.4
*Streptococcus* species	2	5.72 ± 2.6	0.89 ± 0.8

a All values are means ± standard deviations (SD). Values that are significantly different from the value for *S*. *gordonii* are indicated by asterisks as follows: **, *P* < 0.01; ***, *P* < 0.001.

Antagonism toward S. mutans was variable within each species and not correlated with mean ADS activity. We compared the mean antagonistic activity of each species to Streptococcus gordonii for consistency with the ADS activity comparisons and found that S. intermedius and S. sanguinis had significantly lower antagonism than S. gordonii (Fig. 1B and Table 1). S. gordonii and *S. australis*/A12-like isolates all exhibited antagonism toward S. mutans, while S. intermedius was the only species with no isolates that exhibited antagonism, and all other species had isolates with a range of antagonism from none to high (Fig. 1B). The single *S. salivarius* isolate had low ADS activity and was not antagonistic toward S. mutans, while the single *S. parasanguinis* isolate had very high ADS activity and low antagonism. Given the wide range of phenotypes within all other species represented here, it is not possible to speculate on whether these characteristics are representative of *S. salivarius* and *S. parasanguinis*. However, previous studies from our lab have shown that *S. salivarius* is an abundant producer of ammonia via a urease enzyme, which helps to maintain the oral biofilm pH homeostasis (10).

### Distribution of the ADS operon in the genus *Streptococcus*.

To better understand the distribution of the ADS operon within the genus *Streptococcus* and to correlate the presence of the ADS operon within our oral isolates, we manually searched for the operon in a custom-built database of *Streptococcus* RefSeq genomes and performed BLAST searches of the operons found manually against the *Streptococcus* database to look for other genomes with the operon. The entire operon *arcABCDT* and *arcRqueA* were identified in S. constellatus (70%), *S. cristatus* (100%), S. gordonii (96%), S. intermedius (75%), *S. parasanguinis* (70%), and S. sanguinis (97%), all of which are oral species (Table S2). Isolates lacking *arcR* and *queA* were found in S. mitis (3.5%), S. oralis (5.2%), S. oralis subsp. *dentisani* (54%) (9% of S. oralis plus all S. oralis subspecies), S. pneumoniae (94%) and in *Streptococcus* oral taxon 058. The remaining species that had ADS operon genes were S. anginosus (10%), S. canis (100%), S. dysgalactiae (100%), S. merionis (100%), S. pyogenes (78%), and S. uberis (93%) (Table S2), but in all cases, the operon was not contiguous or complete. In some species, the order of the ADS genes had been rearranged, and in others, additional genes or transposons had been inserted without disrupting the genes. Therefore, it remains unclear whether the operon is functional in these species.

10.1128/mSystems.00158-18.2 TABLE S2 List of *Streptococcus* RefSeq genomes used in this study with accession numbers. Download Table S2, XLSX file, 0.03 MB. Copyright © 2018 Velsko et al. 2018 Velsko et al. This content is distributed under the terms of the Creative Commons Attribution 4.0 International license.

### Arginine deiminase activity does not correlate with genotype.

Arginine deiminase activity in *Streptococcus* is governed by the arginine deiminase operon, which includes five structural genes *arcA*, *arcB*, *arcC*, *arcD*, and *arcT*, and the regulatory genes *arcR* and *queA*, which are cotranscribed in the opposite direction from the structural genes (20). The global nitrogen regulator *flp* is also involved in regulating expression of the operon (17), and it was annotated *ntcA* in our genomes. We identified each of these genes in our isolates to compare phylogenetic relatedness with ADS activity. The annotation of these genes was not consistent, sometimes *arcD* and *arcT* were annotated as “hypothetical protein” and “putative dipeptidase,” yet we confirmed a full, contiguous operon and associated regulatory genes as described in Materials and Methods. All eight genes (*ntcA*, *arcABCDT*, *arcR*, *queA*) were present in all isolates of *S. australis*/A12-like, *S. cristatus*, S. gordonii, S. sanguinis and the single *S. parasanguinis* isolate, but were not detected in the *S. salivarius* isolate or the three unidentified species isolates (Table S1). Two of the S. sanguinis isolates have a three-gene insertion between *arcC* and *arcD* that includes *ydgI* and *aspC*, and a duplicated *arcC*, yet this does not appear to have impaired their ADS activity (Table S1). Nine of eleven *S. cristatus* isolates had all eight genes, and the remaining two isolates had none. Very few isolates of S. mitis and S. oralis had any genes in the operon, and when the operon was present, *ntcA* and *arcABCDT* were there, but not *arcR* or *queA*. Six of the 21 S. mitis isolates (29%), one of the eight S. oralis isolates (12%), and five of the six S. oralis subsp. *dentisani* (83%) isolates had this part of the operon (Table S1). The distribution of the ADS operon in our isolates is similar to its distribution in the RefSeq genomes of these species examined above (Table S2). Only one of these genes, *arcD*, tested positive for recombination with phi.

**FIG 2:**
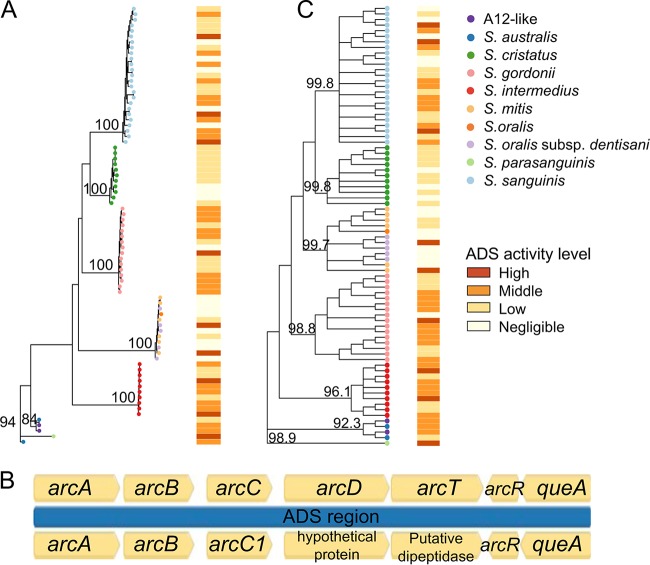
ADS operon genotype and ADS activity level. (A) Maximum likelihood phylogeny of the ADS operon and regulatory elements *arcABCDTRqueA* with a heatmap indicating ADS activity level. (B) Example of the ADS operon and control elements showing protein-coding and intergenic regions used to build the phylogeny in panel A, from S. gordonii strain Challis (top), and an S. gordonii isolate from this study (bottom). Note inconsistencies in the gene annotations. (C) Gene consensus tree of the individual ADS operon gene trees (*arcA*, *arcB*, *arcC*, *arcD*, *arcT*, *arcR*, and *queA*) with a heatmap indicating ADS activity level. Bootstrap values (as percentages) are shown on major nodes.

We built phylogenies of three versions of the full operon region, including all intergenic regions, one *arcABCDT+arcRqueA* (Fig. 2A and B), one *nctA+arcABCDT+arcRqueA* (Fig. S2A and B), and one *arcABCDT* (Fig. S3A and B) to assess the phylogenetic relatedness of the operon and regulatory elements and to determine whether the ADS activity of each isolate is related to genotype. We then built individual phylogenies for each of the eight genes (Fig. S4). The isolates grouped by species in each operon phylogeny and gene consensus trees showed similar branching patterns (Fig. 2C and Fig. S2C and S3C). The branching patterns in each phylogeny closely matched those of the *Streptococcus* genus phylogeny (32). Like the core phylogeny though, the S. mitis, S. oralis, and S. oralis subsp. *dentisani* isolates are intermixed within their own clade. Heatmaps presenting the mean ADS activity for each isolate aligned with the phylogenies (Fig. 2 and Fig. S2, S3, and S4) do not show clear correlations between ADS activity and the species groups or the branching patterns within each species. In the *arcR* phylogeny (Fig. S4 and S5A), the *S. cristatus* isolates split into two groups because the five isolates in the clade more distant to the S. gordonii, S. intermedius, and S. sanguinis isolates have a very short *arcR* sequence. The short *arcR* sequences are genuinely short and not an artifact of assembly such as truncation due to being located at the end of a contig, and removing them from the phylogeny does not alter the branching patterns delineating the species clades (Fig. S5B).

10.1128/mSystems.00158-18.5 FIG S2 ADS operon and regulatory gene *flp*/*ntcA* genotype and ADS activity level. (A) Maximum likelihood phylogeny of the ADS operon *ntcAarcABCDTRqueA* with a heatmap indicating ADS activity level. Bootstrap values (as percentages) are shown on major nodes. (B) Example of the ADS operon and control elements showing protein-coding and intergenic regions used to build the phylogeny in panel A, from S. gordonii strain Challis (top) and an S. gordonii isolate from this study (bottom). Download FIG S2, PDF file, 0.4 MB. Copyright © 2018 Velsko et al. 2018 Velsko et al. This content is distributed under the terms of the Creative Commons Attribution 4.0 International license.

10.1128/mSystems.00158-18.6 FIG S3 ADS operon genotype and ADS activity level. (A) Maximum likelihood phylogeny of the ADS operon *arcABCDT* with a heatmap indicating ADS activity level. (B) Example of the ADS operon showing protein-coding and intergenic regions used to build the phylogeny in panel A, from S. gordonii strain Challis (top) and an S. gordonii isolate from this study (bottom). (C) Gene consensus tree of the individual ADS operon gene trees (*arcA*, *arcB*, *arcC*, *arcD*, *arcT*) with a heatmap indicating ADS activity level. Bootstrap values (as percentages) are shown on major nodes. Download FIG S3, PDF file, 0.4 MB. Copyright © 2018 Velsko et al. 2018 Velsko et al. This content is distributed under the terms of the Creative Commons Attribution 4.0 International license.

10.1128/mSystems.00158-18.7 FIG S4 ADS operon gene phylogenies and ADS activity phenotype. Each individual maximum likelihood gene phylogeny (*ntcA*, *arcA*, *arcB*, *arcC*, *arcD*, *arcT*, *arcR*, and *queA*) is presented adjacent to a heatmap indicating the ADS activity level of each isolate. Download FIG S4, PDF file, 0.3 MB. Copyright © 2018 Velsko et al. 2018 Velsko et al. This content is distributed under the terms of the Creative Commons Attribution 4.0 International license.

10.1128/mSystems.00158-18.8 FIG S5 *arcR* gene phylogenies and ADS activity phenotype. (A) *arcR* phylogeny for all isolates sequenced in this study with a heatmap indicating the ADS activity level of each isolate (same as in Fig. S4). (B) *arcR* phylogeny for all excluding the five *S. cristatus* isolates with short *arcR* sequences, with a heatmap indicating the ADS activity level of each isolate. Download FIG S5, PDF file, 0.2 MB. Copyright © 2018 Velsko et al. 2018 Velsko et al. This content is distributed under the terms of the Creative Commons Attribution 4.0 International license.

### Antagonism of S. mutans does not correlate with known antagonism-related genotypes.

**FIG 3:**
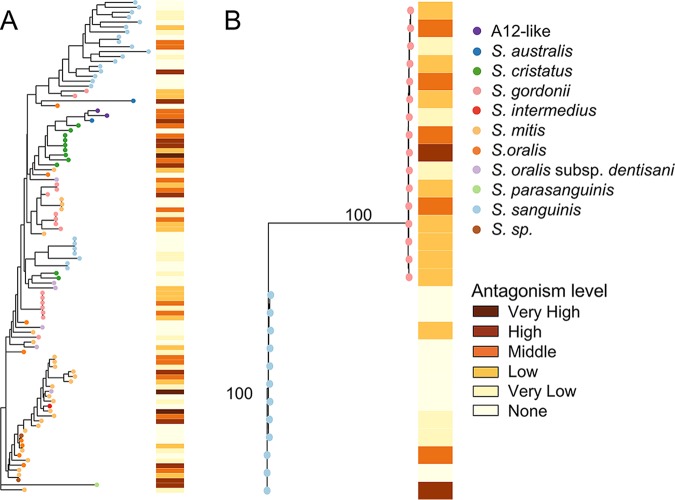
Antagonism-associated genotype and phenotype. (A) Maximum likelihood phylogeny of the pyruvate oxidase gene with a heatmap indicating the level of antagonism toward S. mutans. Bootstrap values were <50% for major nodes. (B) Maximum likelihood phylogeny of the challisin gene with a heatmap indicating the level of antagonism toward S. mutans. Bootstrap values (as percentages) are shown on major nodes. *S*. *sp*., *Streptococcus* species.

It was previously shown that targeted loss of the gene for the H_2_O_2_-generating pyruvate oxidase (*spxB*) or the gene for the serine protease challisin of S. gordonii DL1 and *Streptococcus* A12, which degrades an intercellular signal molecule for S. mutans bacteriocin production, reduces antagonism of these strains toward S. mutans (31), so we examined the phylogenetic relatedness of these genes in our isolates. The pyruvate oxidase gene, annotated *pox5* rather than *spxB*, was present in all isolates of *S. australis*/A12-like, *S. cristatus*, S. gordonii, S. oralis, S. oralis subsp. *dentisani*, *S. parasanguinis*, and S. sanguinis. We confirmed that this gene is equivalent to S. gordonii strain Challis *spxB* by including that gene in our alignment and building a tree that included *spxB* (Fig. S6). All but one S. mitis isolate carried the gene, and both undefined species isolates carried it, while only a single isolate of S. intermedius carried it. The *pox5* phylogeny is not strictly grouped by species like the *arc* gene phylogenies (Fig. 3A), and the gene tested positive for recombination with phi. The majority of S. sanguinis isolates cluster together, and there is a distinct clade of S. mitis/S. oralis/S. oralis subsp. *dentisani*, yet the remaining isolates form mixed-species clades. The heatmap of mean antagonism activity aligned with the tree in Fig. 3A shows no clear relationship between gene phylogeny and antagonistic activity measured in aerobic conditions.

10.1128/mSystems.00158-18.9 FIG S6 Pyruvate oxidase maximum likelihood phylogeny with an antagonism heatmap as in Fig. 3A, but with the S. gordonii strain Challis pyruvate oxidase gene *spxB* included for reference as a black asterisk (15th from the top). Bootstrap values were <50% for major nodes. Download FIG S6, PDF file, 0.06 MB. Copyright © 2018 Velsko et al. 2018 Velsko et al. This content is distributed under the terms of the Creative Commons Attribution 4.0 International license.

The challisin gene was found only in S. gordonii and S. sanguinis isolates and was annotated *scpA*, a C5a protease. The gene was present in all S. gordonii isolates, but it was found in only 12 of 25 S. sanguinis isolates (Fig. 3B). In the phylogeny, the isolates cluster by species, and the short branches within the species show that there is very little variation in the gene sequences. It has not been shown that challisin itself has antagonistic activity, but it might enhance antagonism by diminishing the amount of bacteriocins that S. mutans can produce, thereby allowing for better growth and production of antagonistic factors by the commensal. However, although more S. gordonii isolates have high antagonism than do S. sanguinis isolates, the heatmap of antagonism shows no clear correlation between the challisin phylogeny and antagonism activity. Like *pox5*, *scpA* tested positive for recombination with phi.

### Genus- and species-specific genes potentially involved in ADS activity and antagonism.

To search for additional genes that may be involved in ADS activity or S. mutans antagonism, we screened our isolates using two approaches to detect bacterial pan-genome–phenotype association. We searched for genes associated with these phenotypes across all of our isolates, as well as within each of the species groups. Using the first approach (Scoary), no significant associations between gene clusters and phenotype were found when running 100 permutations and a Benjamini-Hochberg corrected *P* value cutoff of 0.05. In contrast, using the second approach (treeWAS), we found sets of genes significantly associated with ADS activity and antagonism across all species, as well as within S. mitis, S. oralis, and S. sanguinis (Table S3). The difference in the statistical approaches between these two programs may explain our contrasting findings: treeWAS compares associations between a null data set and the real one, while Scoary performs multiple test corrections, which can be a severe approach that removes true-positive hits (33, 34). Several of the genes associated with ADS activity in all *Streptococcus* isolates we sequenced are involved in arginine processing, including arginine transport system permease *artQ*, arginine decarboxylase, and arginine-binding extracellular protein *artP* precursor (Table S3), while several others were involved in outer membrane transport or other seemingly unrelated processes or were hypothetical proteins. Fewer genes were associated with antagonism in all *Streptococcus* isolates than with ADS activity, and they included DNA-binding transcriptional repressor *acrR*, a type 1 restriction enzyme R protein, and a bacteriophage holin.

10.1128/mSystems.00158-18.3 TABLE S3 treeWAS results. Download Table S3, XLSX file, 0.01 MB. Copyright © 2018 Velsko et al. 2018 Velsko et al. This content is distributed under the terms of the Creative Commons Attribution 4.0 International license.

None of the genes associated with ADS activity or antagonism in the full set of isolates were identified in any of the species-specific tests for association. S. oralis and S. oralis plus S. oralis subsp. *dentisani* both had a single gene associated with antagonism, *amiA1* encoding oligopeptide-binding protein AmiA, which was also identified in the S. mitis genome-wide association study (GWAS) (Table S3). S. sanguinis had six genes associated with ADS activity, three of which were hypothetical proteins. One gene, annotated carbamoyl phosphate synthase-like protein, is involved in arginine metabolism, while the relation of the remaining two annotated genes, enterobactin exporter EntS and UDP-*N*-acetylglucosamine 1-carboxyvinyltransferase 2, to ADS activity is not clear (Table S3). A single gene, transcriptional regulator *mtrR1*, was associated with antagonism in S. sanguinis. None of the remaining species groups had any genes significantly associated with ADS activity or antagonism.

## DISCUSSION

We performed a genome-wide study of a phylogenetically and phenotypically diverse set of oral streptococci isolated from health-associated supragingival dental plaque to characterize the genotypic basis of variation in ADS activity and antagonism of S. mutans. We demonstrated that these two phenotypes vary substantially within and between species, yet the phylogenetic relationship of the genes associated with these phenotypes through earlier studies do not reflect the actual phenotypes. Our results support the observation (18) that differences in transcriptional or translational control may influence the expression of genes responsible for these phenotypes more than the gene sequences themselves.

The ADS operon genes are widely distributed in *Streptococcus*, but they appear to be maintained as a contiguous (and presumably functional) operon predominantly in the oral *Streptococcus* species. This may be directly related to their lifestyle in oral biofilms, which are frequently acidified by other biofilm species, in contrast to other *Streptococcus* species such as S. pyogenes or *S. uberis*, which are not known to inhabit dense biofilms that are commonly subjected to frequent acidification. However, the operon is clearly functional in the *Streptococcus* species we screened when the genes are not contiguous or when lacking regulatory genes. Insertion of three genes between *arcC* and *arcD* in two S. sanguinis strains was not associated with diminished ADS activity, and despite the S. mitis or S. oralis strains with the operon missing *arcR* and *queA,* AD activity is still expressed. While the average ADS activities for S. mitis and S. oralis are lower than the activities of other strains, the lack of *arcR* and *queA* does not necessarily explain this, as several strains of S. gordonii, *S. cristatus*, and *S. salivarius* have these regulatory genes yet have low ADS activity.

Regulation of the ADS operon is complex, and it is not surprising that there is no clear relationship between operon genotype and ADS activity. Expression can be repressed by oxygen, enhanced at low pH, and increased by arginine concentration, and it involves several regulatory genes, carbohydrate catabolite repression, and two-component systems (20). Many genes are involved in ADS pathway activation, and this network of regulation may determine expression levels that are unrelated to the sequences of the structural genes. This regulatory network can be identified by functional studies, but not genomic studies alone. In addition, posttranscriptional control of the ADS operon may be important in determining expression levels (20), which again cannot be captured by genomic surveys.

The phylogenetic relationships of antagonism-associated genes such as the genes encoding pyruvate oxidase, which produces H_2_O_2_ that inhibits S. mutans directly, and challisin, which interferes with S. mutans bacteriocin production potentially reducing fitness of S. mutans, within the isolates that we sequenced do not correlate with the antagonism phenotypes of each isolate, just as we saw for ADS activity genotype and phenotype. There are some clusters of species within the *pox5* pyruvate oxidase phylogeny, but the species groups are much more mixed than was seen with any of the ADS operon genes, which suggests that this gene may be subject to horizontal transfer. The *pox5* gene tested positive for recombination with phi, which supports horizontal transfer between *Streptococcus* species. Similar to the ADS operon gene phylogenies, there is no clear correlation between *pox5* genotype and antagonism phenotype, with the exception of S. intermedius. None of the S. intermedius isolates were antagonistic toward S. mutans, and only a single isolate had the *pox5* gene. Although the challisin gene shows a distinct species-related phylogenetic signal, it shows no correlation with antagonism phenotype. The indistinct relationships between pyruvate oxidase genotype and antagonism as well as challisin genotype and antagonism may again be related to the transcriptional, translational, and/or posttranslational control of these genes, or in the case of challisin to differences in the substrate specificity of the enzyme.

Our genome-wide association studies did not report associations between ADS activity or antagonism and the genes involved in these phenotypes for which we built phylogenies. Given the complex network regulating ADS operon expression discussed above, this is not surprising. However, several genes that were identified by treeWAS as significantly associated with ADS activity are involved in arginine processing, and therefore, the genes identified by this method should be investigated by functional studies for their role in arginine processing and ammonia production. Our small sample size, especially for the individual species groups, may prevent us from finding significantly associated genes, and these GWAS studies should be performed with more isolates to obtain better power, particularly if functional interrelationships can be established with the gene products we identified using treeWAS and ADS levels.

A single isolate each of *S. salivarius* and *S. parasanguinis* and two isolates similar to the recently described strain A12 (31) based on 16S rRNA gene similarity were included in our analysis. *S. salivarius* is the most distantly related of the *Streptococcus* species we included in this study (32), and the isolate we sequenced did not contain any ADS operon genes. None of the 44 RefSeq *S. salivarius* genomes we screened had the ADS operon, so this species may rely instead on the urease gene cluster to produce ammonia to counter drops in pH (10). However, the full urease operon (35) was present only in our *S. salivarius* isolate and not in any of our other isolates, based on a BLAST search we performed. Urease activity is higher in plaque from caries-free adults than in plaque from adults with caries (27), so this pathway may be desirable in probiotic strains, yet it may be restricted to specific oral species such as *S. salivarius*, Actinomyces naeslundii, and *Haemophilus* (36). More *S. salivarius* strains will need to be characterized for ammonia production and S. mutans antagonism to understand the range of ammonia production in this species and its potential as a probiotic. In contrast, the *S. parasanguinis* isolate had high ADS activity and moderately antagonized S. mutans, and the range of activity in this species should also be further investigated.

*S. australis* and the A12-like isolates, which are phylogenetically closely related (31), have moderate to high ADS activity and S. mutans antagonism. This finding supports earlier conclusions that A12-like isolates may make an excellent probiotic candidate (31). The A12-like isolates are rare, and our plaque screens identified only two, both of which we included in this study. What the infrequent isolation of A12-like organisms means for the ecology of this organism in the mouth and plaque biofilm is uncertain, and the ability of this organism to integrate and be maintained in the oral biofilm of patients who do not naturally carry it will need to be studied. Unfortunately, a retrospective examination of microbiome studies that used 16S rRNA gene sequencing would not be informative, as the 16S rRNA genes of A12-like organisms, *S. australis,* and *S. parasanguinis* share 99% identity and cannot be easily distinguished. Whether A12-like isolates are strains of *S. australis* or a distinct species is unclear from our core and gene phylogenies. We are in the process of obtaining, characterizing, and sequencing more A12-like isolates to clarify the relationship between this species and *S. australis* and its placement in the phylogeny of the genus *Streptococcus*.

In sum, we have shown that the extensive variation in ADS activity and S. mutans antagonism within oral *Streptococcus* species cannot be solely explained by genotypic variation. Complex regulation of these phenotypes may explain the differences within and between species but cannot be assessed by gene sequence analysis or genome-wide surveys. To develop probiotics that take advantage of ammonia production and growth inhibition of S. mutans, strains will need to be carefully selected based on laboratory screening and phenotypic characterization and not on species designation alone.

## MATERIALS AND METHODS

### Plaque collection and bacterial strain isolation.

Supragingival dental plaque was collected from both children (*n* = 29) and adults (*n* = 11) who were caries free individuals, having no clinical evidence of present or prior dental caries activity (decayed, missing, and filled teeth [DMFT] = 0). Informed consent was obtained from all participating subjects (parents gave consent for their children) under reviewed and approved protocols by the Institutional Review Board of the University of Florida Health Science Center (approval number IRB201600154 for children’s study and IRB201600297 for adult study). Children and adult individuals were required to refrain from oral hygiene procedures for 8 and 12 h prior to the collection of dental plaque, respectively. Plaque samples were collected from the surfaces of teeth using sterile periodontal curettes, then transferred to sterile, chilled microcentrifuge tubes containing 10 mM sodium phosphate buffer (pH 7.0), and stored at −80°C until further analysis. To isolate cultivable oral *Streptococcus* species (27, 29), plaque samples were dispersed by external sonication (FB120; Fisher Scientific, Hampton, NH, USA) for two cycles of 15 s with 30-s cooling on ice between the two cycles. Portions (10 µl) of the dispersed plaque samples were then serially diluted in 10 mM sodium phosphate buffer (pH 7.0), and 100-µl portions of the diluted samples (10^−4^ to 10^−7^) were plated on sheep blood agar (Columbia agar base containing 5% [vol/vol] of anticoagulated sheep blood; Difco Laboratories, MI, USA) and on BHI (Difco Laboratories) agar. The plates were placed in anaerobic jars (BBL GasPak systems, BD Diagnostics, MD, USA) and incubated at 37°C for 48 h. Colonies of all clinical isolates from both blood agar and BHI agar plates were collected and further subcultured on the same media and incubated subsequently in a 5% CO_2_ aerobic incubator until pure colonies were obtained.

### Preliminary species identification by 16S rRNA gene sequencing.

To select only *Streptococcus* isolates for biochemical characterization, we sequenced the 16S rRNA gene of our clinical isolates to assign each to a species. An optimized PCR using a universal primer set (forward, 5′-AGA GTT TGA TCC TGG CTC AG-3′; reverse, 5′-TAC GGG TAC CTT GTT ACG ACT-3′) was used to amplify the full 16S rRNA gene from each clinical isolate (37). PCR products were then cleaned using a Qiaquick PCR cleanup kit (Qiagen, Valencia, CA, USA) and sequenced by Sanger sequencing at the University of Florida Interdisciplinary Center for Biotechnology (UF-ICBR) for primary identification of isolated bacterial species. A putative species designation for each isolate was determined by a nucleotide BLAST search using the online BLAST search engine at NCBI with default parameters against the 16S rRNA sequences (bacteria and archaea) database, and the hit with the highest bit score was selected.

### ADS activity.

All isolated clinical oral streptococci (a total of 113 isolates) were tested for their potential to generate citrulline from arginine via the arginine deiminase system (ADS) by a protocol previously validated and published by our group (20). Briefly, a single colony of each clinical isolate was inoculated in tryptone-yeast extract (TY) broth containing 25 mM galactose and 10 mM arginine and incubated overnight at 37°C in a 5% CO_2_ aerobic incubator. Overnight cultures were then diluted (1:20) in the same media until exponential phase (optical density at 600 nm [OD_600_] of 0.5 to 0.6). The cells were harvested, washed, and resuspended in 10 mM Tris-maleate buffer and further permeabilized with toluene-acetone (1:9) for the measurement of ADS activity. The total protein concentration of the cell suspension was also measured by using BCA protein estimation kit (Pierce, Waltham, MA, USA) with a known concentration of bovine serum albumin (BSA) as the standard. The ADS activity levels in the clinical isolates were normalized to protein content and represented as nanomoles of citrulline generated per minute per milligram of protein. Streptococcus gordonii DL1 was used as the reference strain for this assay.

### Competition assay.

BHI agar plates were used for competition assays between commensal streptococci and oral pathogen Streptococcus mutans UA159. Overnight cultures from single colonies were adjusted to an OD_600_ of 0.5. Six microliters of each culture was then spotted adjacent to each other on agar plates with the commensal spotted first and strain UA159 spotted 24 h later. All experiments were performed under aerobic conditions. ImageJ software was used to measure the zone of inhibition (in millimeters) between competing colonies on the plate.

### Statistical analysis.

Statistical differences in mean ADS activity and mean S. mutans antagonism were calculated by one-way ANOVA using S. gordonii as the reference group with Bonferroni multiple-test correction in Prism v7.0d. Graphs were generated using Prism v7.0d.

### DNA isolation and Illumina sequencing.

For whole-genome shotgun sequencing, genomic DNA was isolated from each commensal *Streptococcus* using Wizard Genomic DNA purification kit (Promega, Madison, WI, USA) with some modifications. Briefly, 8-ml overnight culture of each isolate was harvested and resuspended in 480 µl of EDTA, and appropriate lytic enzymes were added to the cell suspension (100 µl of 10 mg/ml lysozyme and 2 µl of 5 U/µl of mutanolysin). Cells were harvested after an incubation of 1 h at 37°C. Then 600 µl of nuclei lysis solution (provided by the manufacturer) was added to the cell suspension, and the samples were incubated at 80°C for 5 min. This step was necessary for the breakdown of the cell wall. RNase was added to cell lysate and incubated about an hour at 37°C to inhibit RNA contamination while purifying genomic DNA. To minimize protein impurities, protein precipitation solution (provided by the manufacturer) was added to the RNase-treated cell lysate, mixed by vigorous vortexing, and incubated on ice for 5 min. The total cell lysate was then harvested, and the supernatant containing the DNA sample was transferred to a fresh tube containing room temperature isopropanol. The supernatant was rotated at room temperature about an hour or until the thread-like strands of DNA formed a visible mass. Finally DNA was purified in nuclease-free water after two washes in 70% ethanol. Total DNA concentration was measured using NanoDrop One Microvolume UV-Vis spectrophotometer (ThermoFisher Scientific, Waltham, MA, USA), and DNA integrity was determined by 260/280 ratio. DNA from each isolate was prepared for next-generation whole genome shotgun sequencing using 2 to 5 ng of DNA and the Illumina Nextera-XT library preparation and indexing kit. Libraries were built without deviation from the Illumina recommended protocol, but they were normalized by hand, and not with the beads provided in the Nextera-XT kit. Libraries were pooled at a final concentration of 2 nM and sequenced on an Illumina MiSeq using the Illumina MiSeq v2 kit with paired-end sequencing and 250-bp reads. Reads were demultiplexed by the Illumina software, and the raw fastq files were further processed for analysis.

### Read processing, assembly, and annotation and gene clustering.

Estimated coverage of each genome was calculated by multiplying the number of reads in each raw fastq file by the read length (250 bases) and then dividing by the average number of nucleotides in a *Streptococcus* genome (2.9 Mbp). Coverage ranged from 20× to 300×. The reads were quality trimmed, the genomes were assembled using the program A5 (38) with default parameters, and assembly quality was assessed with quast v4.6.3 (39). Assembled genomes were annotated with Prokka v 1.11 (40) using a *Streptococcus*-specific amino acid gene sequence database. For gene clustering, Prokka-annotated amino acid fasta files for the isolates we sequenced along with the Streptococcus mutans files were concatenated into one file, as well as the arginine deiminase genes *arcA*, *arcB*, *arcC*, *arcD*, *arcT*, and *arcR* from Streptococcus gordonii strain Challis (NCBI accession CP000725.1) for easy identification of these genes during analysis. Homologous genes among all genomes were delineated using the MCL algorithm (41) as implemented in the MCLBLASTLINE pipeline (available at http://micans.org/mcl). The pipeline used Markov clustering (MCL) to assign genes to homologous clusters based on an all-vs-all BLASTX search with DIAMOND v0.8.22.84 (42) between all pairs of protein sequences using an E value cutoff of 1e−5. The MCL algorithm was implemented using an inflation parameter of 1.8. Simulations have shown this value to be generally robust to false-positive and false-negative results (43).

### Species identification by core genome phylogeny.

For comprehensive identification, a core genome of single-copy genes present in all isolates we sequenced was determined from the MCL clustering. A total of 608 single-copy core gene clusters were identified, and these were aligned using MUSCLE (44) and checked for recombination using PhiPack (45). Genes identified as recombinant by all three tests (phi, NSS, max χ^2^) were removed from the core gene group. The remaining 425 putatively nonrecombinant single-copy core gene alignments were concatenated, and the concatenated alignment was used to build a core phylogeny using phyML v. 3.0 (46) with the GTR+G substitution model. Bootstrap support was provided by generating 100 bootstrap replicates. The species designations for all isolates were compared between the 16S rRNA gene and core gene phylogeny, and several discrepancies were found. All isolates were assigned to a species based on the core gene phylogeny.

### Distribution of the ADS operon in the genus *Streptococcus*.

To determine the distribution of the contiguous ADS operon within the genus *Streptococcus* and our sequenced isolates, we built a comprehensive *Streptococcus* custom BLAST database using the software Geneious v7.0 (Biomatters Inc., Newark, NJ, USA). The database was built using GenBank files from RefSeq at NCBI and those generated by Prokka for our isolates. Consequently, the database contained assembled and annotated contigs, and information regarding gene synteny was available. All RefSeq *Streptococcus* genomes were downloaded from NCBI on 16 April 2018. Fifty genomes of S. agalactiae, S. equi, S. pyogenes, S. pneumoniae, S. suis, and unidentified *Streptococcus* species were randomly selected for inclusion in the database, as there are many more entries of these species in NCBI than the other *Streptococcus* species. Half of the Streptococcus mutans genomes (94 of 187) were included in the database, and all of the S. oralis (85) and S. mitis (57) genomes were included because we were particularly interested in distribution of the ADS operon in oral *Streptococcus*. We used a total of 1,083 *Streptococcus* genomes (Table S3) to build the database with the Geneious v7.0 software program. To obtain a BLAST search query sequence of the contiguous operon, we used Geneious to manually search for the *arcA* gene within the genome sequence of S. gordonii strain Challis ADS. This procedure located the operon within a genome and allowed extraction of its contiguous nucleotide sequence.

### Identification of genes involved in the arginine deiminase system.

Gene clusters representing genes in the ADS operon (*arcA*, *arcB*, *arcC*, *arcD*, *arcT*) were identified by the presence of S. gordonii strain Challis ADS pathway genes in those clusters. The sequences were extracted from the Prokka-annotated fasta files of each isolate by locus tags. We confirmed that each gene was part of the ADS operon in each isolate and not a homologous anabolic counterpart by checking that the locus tag for each gene was sequential with the other ADS operon gene locus tags, and we also confirmed that the locus tags of individual genes matched those of the full operon sequence. The *arcR* and *queA* genes were confirmed by checking that the locus tags were sequential with and immediately downstream of the ADS operon, while the regulator *flp* was confirmed by checking that the locus tag was sequential with and immediately upstream of the ADS operon.

The full operon and regulatory genes were identified in our sequenced isolates by manually searching for the operon in a randomly selected representative isolate of each species and performing a BLAST search in Geneious as follows: the annotated genome of one isolate of each species was searched for the *arcA* annotation, and the full operon with regulatory genes *flp*, *arcR*, and *queA* was selected and extracted. The extracted full operon was used as the query in a BLAST search against all of our sequenced isolates.

### Identification of genes involved in antagonism and the urease operon.

Homologous gene clusters representing the serine protease challisin and the pyruvate oxidase *spxB* were identified by a BLAST search using these genes from S. gordonii strain Challis as queries against the genomes of all isolates we sequenced. Homologues of S. gordonii strain Challis pyruvate oxidase gene *spxB* were annotated *pox5* in our isolates. We manually searched for the urease operon in our single *S. salivarius* isolate in Geneious and extracted the full operon region as we did for the ADS operon. We used this as the search query for a BLAST search against all of our sequenced *Streptococcus* isolates to determine whether the urease operon was present in any of our isolates.

### Association of phenotype and genotype using known genes.

Each gene cluster (protease challisin and *spxB*) as well as the full ADS operon was aligned using MAFFT (47) in Geneious, and a phylogeny was generated using phyML with the GTR substitution model and SPR branch swapping. Branch support was generated via 100 bootstrap replicates. Then, a phylogeny based on the consensus of the separate phylogenies for each gene (gene trees) (*arcA*, *arcB*, *arcC*, *arcD*, *arcT*, *arcR*, and *queAi*) was constructed using the triple construction method as implemented in the program Triplec (48) (10,000 iterations). This procedure is based on the observation that the most probable three-taxon tree consistently matches the species tree (49). The method searches all input trees for the most frequent of the three possible rooted triples for each set of three taxa. Once found, the set of rooted triples are joined to form the consensus tree using the quartet puzzling heuristic (50). The method has been shown to outperform majority-rule and greedy consensus methods (51). All phylogenies were graphed using the R package ggtree (52). In addition, the alignment for each gene cluster was tested for recombination with PhiPack (37).

### Pan-genome–phenotype association.

We searched for genes associated with the phenotypes for our isolates using two genome-wide association approaches: Scoary (34) and treeWAS (33). For Scoary, the genomes of each species were clustered independently using Roary (53) and combined with binary coding of the phenotypes. treeWAS was run using both the individual species clustering obtained from Roary and combined species clustering obtained using MCLblastline. The phenotypes for treeWAS were coded as both binary and continuous (see Table S1 in the supplemental material).

### Data availability.

The raw reads from all genomes we sequenced for this study are available to download from the NCBI SRA under accession number PRJNA480251, and the assembled, annotated files are available, as the whole-genome shotgun project has been deposited at DDBJ/ENA/GenBank under the same accession number. Accession numbers for the raw reads and assemblies of each sample are listed in Table S1.
