# Supplementary figures and images for: Species Designations Belie Phenotypic and Genotypic Heterogeneity in Oral Streptococci
Source: mSystems. 2018 Dec 18;3(6):e00158-18. doi: 10.1128/mSystems.00158-18 (PMC6299155; doi:10.1128/mSystems.00158-18)

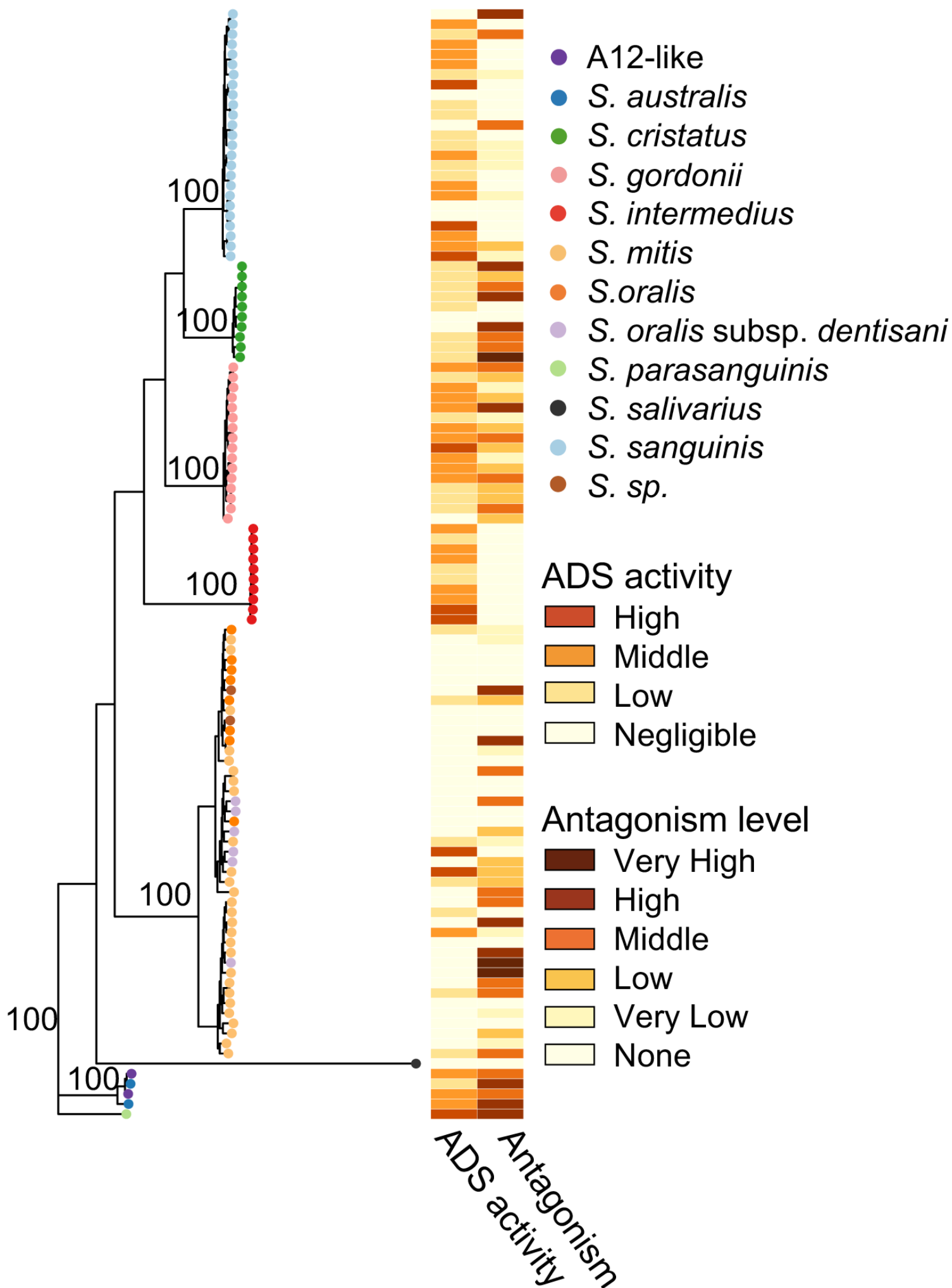

Supplement: FIG S1 [file sys006182304sf1.pdf]

A

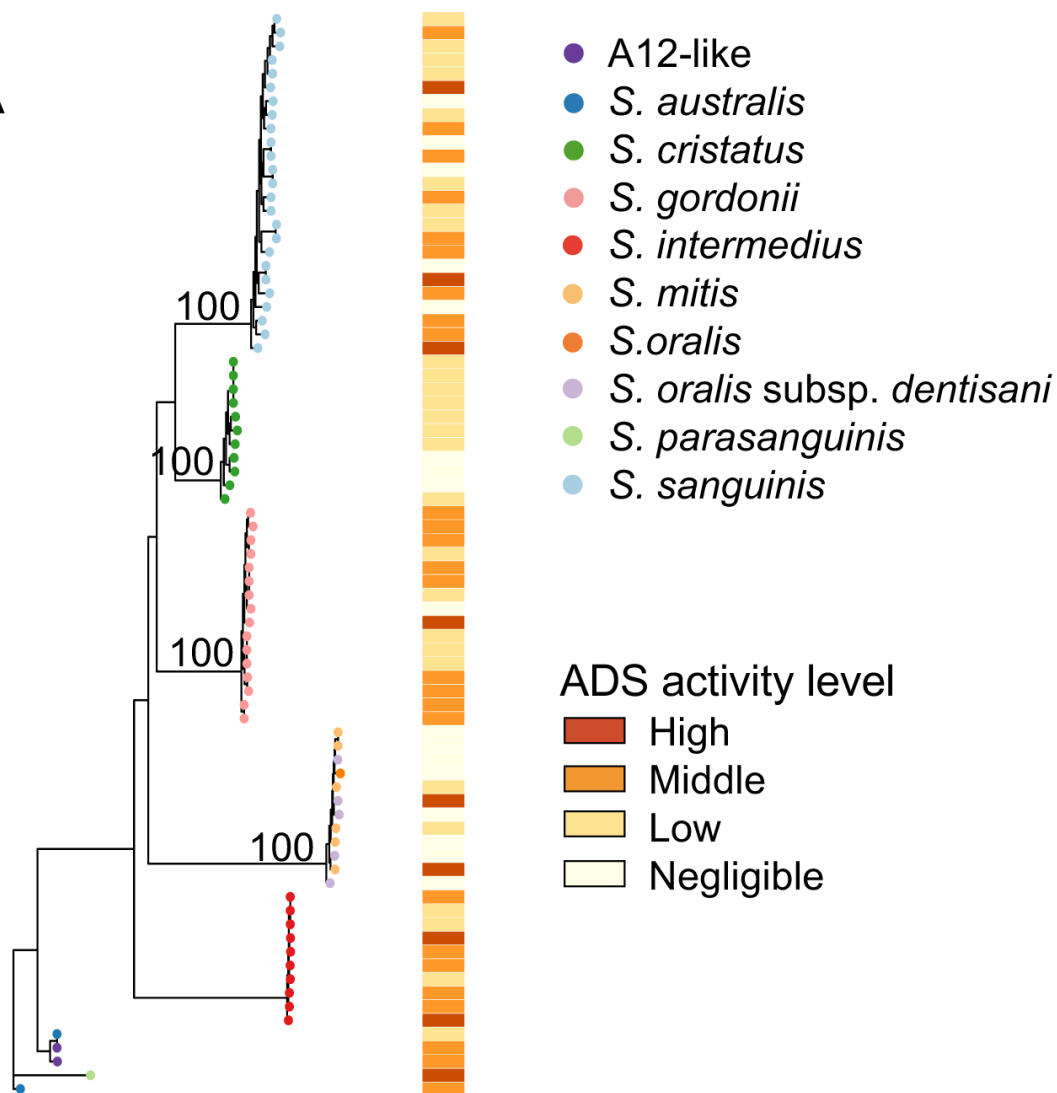

B

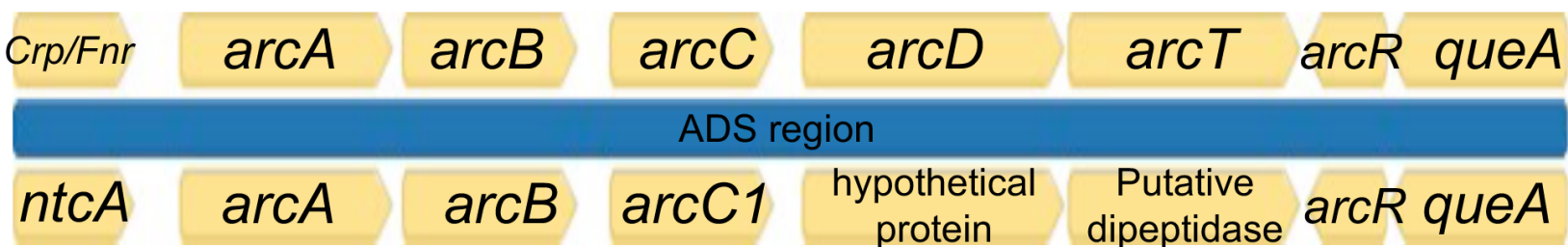

Supplement: FIG S2 [file sys006182304sf2.pdf]
