# Supplementary material for: Species Designations Belie Phenotypic and Genotypic Heterogeneity in Oral Streptococci
Source: mSystems. 2018 Dec 18;3(6):e00158-18. doi: 10.1128/mSystems.00158-18 (PMC6299155; doi:10.1128/mSystems.00158-18)
Supplement: FIG S3 [file sys006182304sf3.pdf]

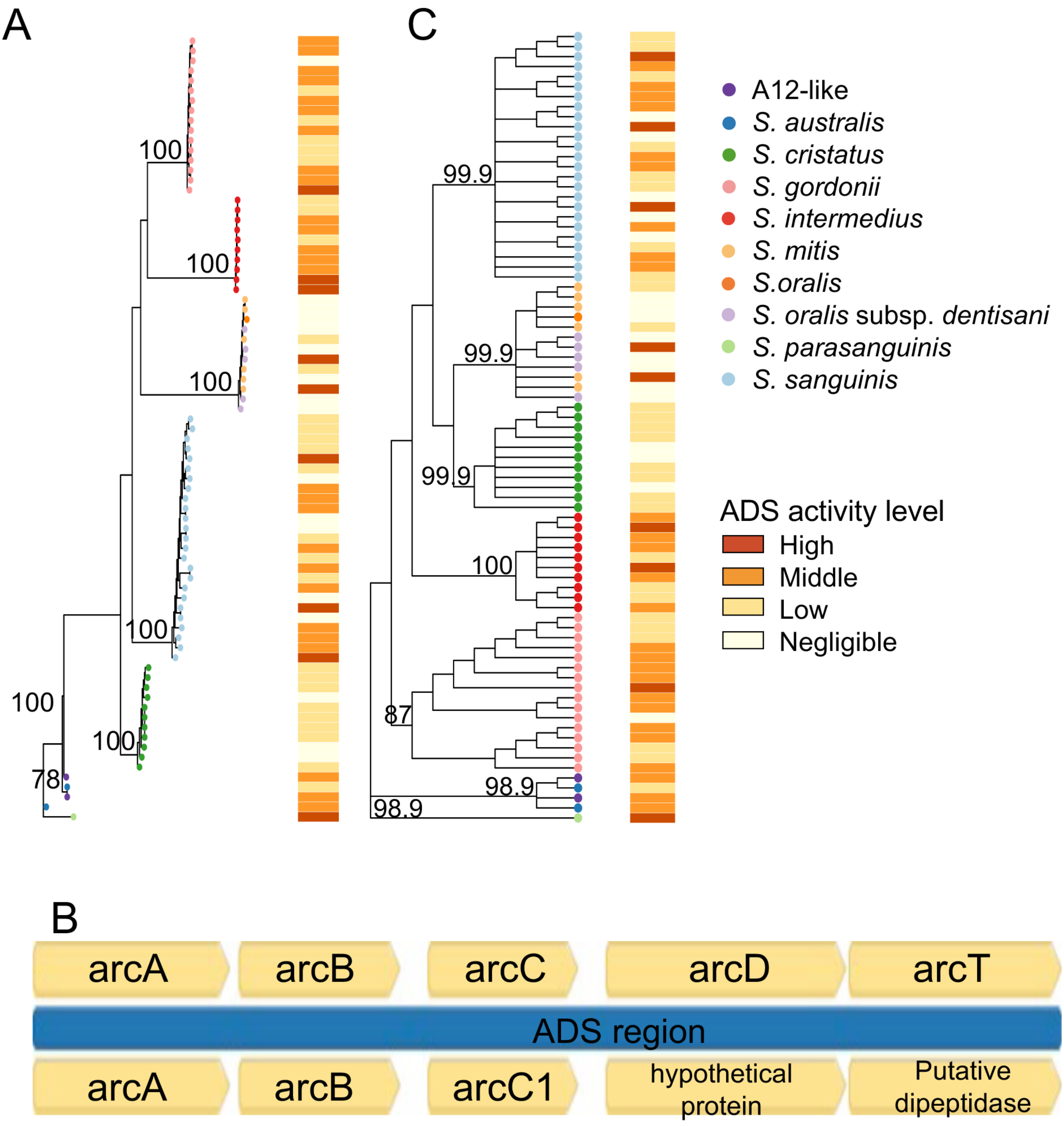
A
100
100
100
100
100
100
78
C
99.9
99.9
99.9
100
87
98.9
98.9
A12-like
S. australis
S. cristatus
S. gordonii
S. intermedius
S. mitis
S.oralis
S. oralis subsp. dentisani
S. parasanguinis
S. sanguinis
ADS activity level
High
Middle
Low
Negligible
B
arcA
arcB
arcC
arcD
arcT
ADS region
arcA
arcB
arcC1
hypothetical protein
Putative dipeptidase
