# Supplementary material for: Species Designations Belie Phenotypic and Genotypic Heterogeneity in Oral Streptococci
Source: mSystems. 2018 Dec 18;3(6):e00158-18. doi: 10.1128/mSystems.00158-18 (PMC6299155; doi:10.1128/mSystems.00158-18)
Supplement: FIG S5 [file sys006182304sf5.pdf]

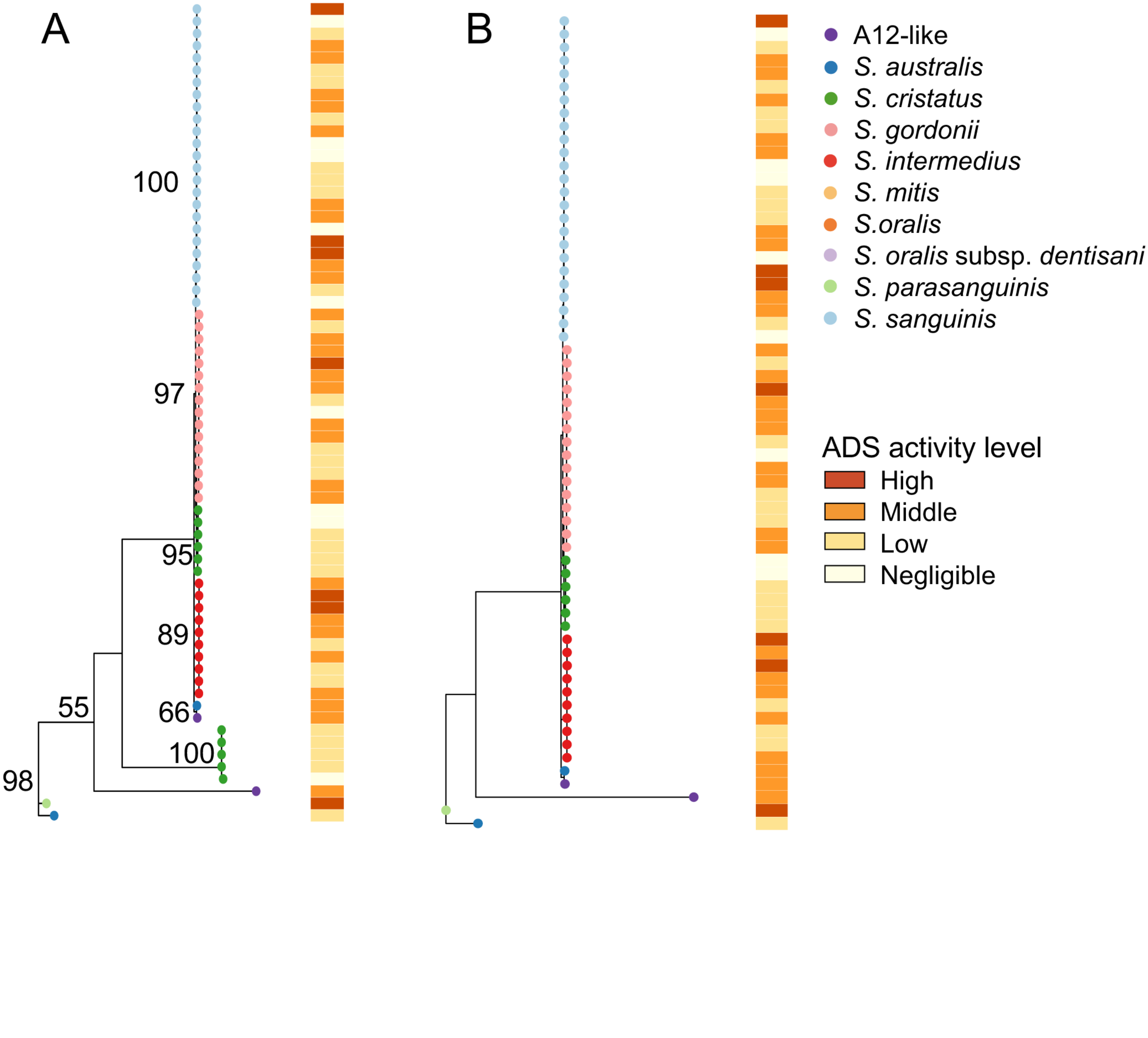
A
B
100
97
95
89
66
55
100
98
A12-like
S. australis
S. cristatus
S. gordonii
S. intermedius
S. mitis
S.oralis
S. oralis subsp. dentisani
S. parasanguinis
S. sanguinis
ADS activity level
High
Middle
Low
Negligible
